# Supplementary material for: Tumor BRCA Testing in Epithelial Ovarian Cancers: Past and Future—Five-Years’ Single-Institution Experience of 762 Consecutive Patients
Source: Cancers (Basel). 2022 Mar 23;14(7):1638. doi: 10.3390/cancers14071638 (PMC8996829; doi:10.3390/cancers14071638)
Supplement: Supplementary file 1 [file cancers-14-01638-s001.zip › cancers-1611965-supplementary.pdf]

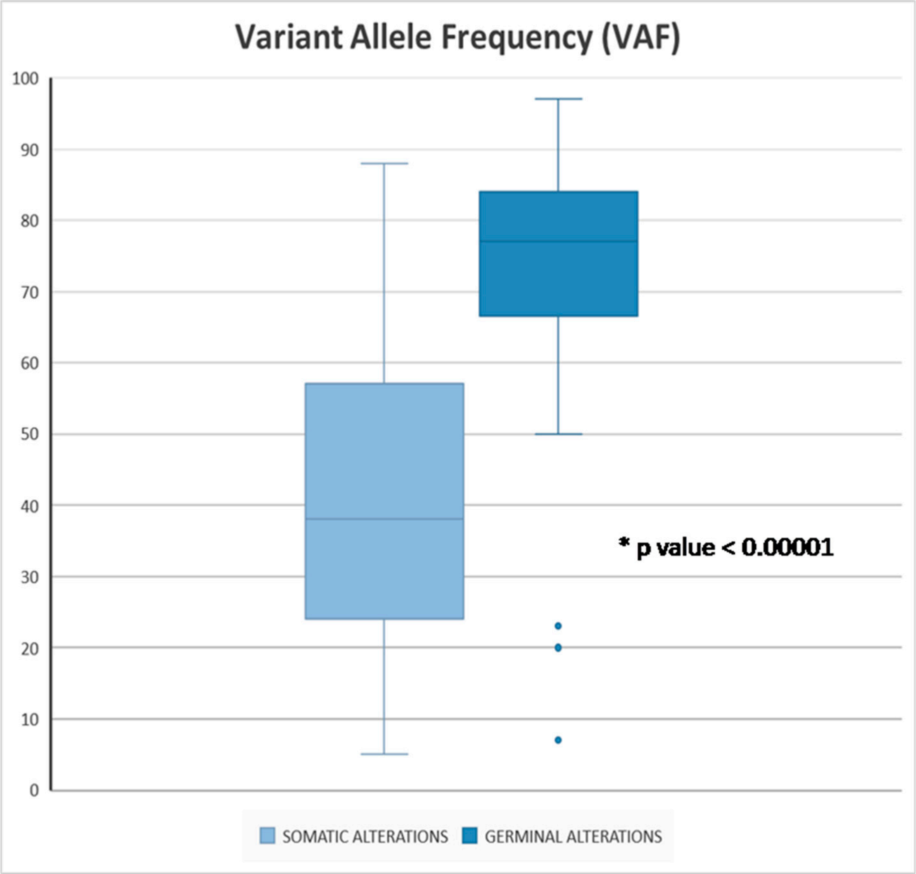

Figure S1 Supplementary: Variant Allele Frequencies (VAF) of somatic and germinal alterations. A statistically significant difference among VAF distribution was reported according to the somatic or germline nature of the alteration.
